# The utility of serum amyloid A and other acute-phase reactants determination in ambulatory care COVID-19 patients

**DOI:** 10.5937/jomb0-42799

**Published:** 2023-08-25

**Authors:** Boris Jegorović, Aleksandra Nikolić, Neda Milinković, Svetlana Ignjatović, Sandra Šipetić-Grujičić

**Affiliations:** 1 University Clinical Center of Serbia, Clinic for Infectious and Tropical Diseases "Prof. Dr. Kosta Todorović", Belgrade; 2 University of Belgrade, Faculty of Medicine, Institute for Epidemiology, Belgrade; 3 University of Belgrade, Faculty of Pharmacy, Department of Medical Biochemistry, Belgrade

**Keywords:** serum amyloid A, SAA, COVID-19, SARSCoV-2, ambulatory care, acute-phase reactants, serum amiloid A, SAA, COVID-19, SARSCoV-2, ambulantno lečenje, reaktanti akutne faze

## Abstract

**Background:**

The unpredictable course of Coronavirus Disease 19 (COVID-19) is making good severity assessment tools crucial. This study aimed to assess the usefulness of serum amyloid A (SAA) and other acute-phase reactants (APRs) in ambulatory care COVID-19 patients and identified relationships between these markers and disease outcomes.

**Methods:**

From August to November 2020, patients seen in the outpatient department of the Clinic for Infectious and Tropical Diseases (Belgrade, Serbia) with confirmed COVID-19 were included. Patients were classified into mild, moderate, and severe disease groups based on World Health Organization criteria. SAA, C-reactive protein (CRP), interleukin-6 (IL-6), procalcitonin (PCT), ferritin, fibrinogen, D-dimer, albumin, and transferrin were measured. The median values of all APRs were compared between COVID-19 severity groups, hospitalized and non-hospitalized patients, and survivors and non-survivors. The Receiver operator characteristic (ROC) curve analysis was used for the classification characteristics assessment of individual APRs for the severity of illness, hospitalization, and survival.

## Abstract (sr)

**Uvod:**

Tok koronavirusne bolesti 2019 (COVID-19) je nepredvidiv, zbog čega su neophodni dobri alati za procenu težine ove infekcije. Svrha ovog istraživanja je procena značaja određivanja serumskog amiloida A (SAA) i drugih reaktanata akutne faze (RAF) kod ambulantnih pacijenata sa COVID-19, kao i identifikacija veze između ovih markera i ishoda bolesti.

**Metode:**

Od avgusta do novembra 2020. godine, u studju su uključeni pacijenti koji su pregledani u Prijemnoj ambulanti Klinike za infektivne i tropske bolesti (Beograd, Srbija), sa potvrđenim COVID-19. Pacijenti su bili klasifikovani u grupe blage, umerene i teške bolesti na osnovu kriterijuma Svetske zdravstvene organizacije. Određivane su vrednosti SAA, C-reaktivnog proteina (CRP), interleukina-6 (IL-6), prokalcitonina (PCT), feritina, fibrinogena, D-dimera, albumina i transferina. Medijane vrednosti RAF su poređene između grupa težine bolesti, bolesnika koji su bili hospitalizovani i onih koji nisu, kao i preživelih i preminulih. Korišćena je analiza Receiver operator characteristic (ROC) krive i Area-under-curve (AUC) za analizu klasifikacionih karakteristika pojedinačnih RAF za težinu bolesti, hospitalizaciju i preživljavanje.

**Rezultati:**

Viši nivoi SAA, CRP, IL-6, PCT i niži nivoi transferrina i albumina su uočeni kod osoba sa teškim oblikom bolesti, hospitalizovanih pacijenata i preminulih. Na osnovu analize ROC krive, AUC za SAA je pokazao zadovoljavajuće klasifikacione performanse za težinu bolesti (0,794) i smrtni ishod (0,732) i dobre performanse za hospitalizaciju (0,853).

**Zaključak:**

SAA je vredan marker u svakodnevnoj praksi za procenu težine i prognoze COVID-19 kod ambulantnih pacijenata.

## Introduction

Coronavirus Disease 19 (COVID-19) is a new viral infectious disease caused by Severe AcuteRespiratory Syndrome Coronavirus-2 (SARS-CoV-2), which first emerged in late December 2019 in China in the city of Wuhan [1]. This new disease spread quickly worldwide, and on March 11, 2020, the World Health Organization (WHO) pronounced the pandemic [2]. COVID-19 can have an extensive range of possible clinical manifestations, from asymptomatic infection to a fatal illness, with the tendency of seemingly mild illness to become more severe in some patients [3]. Considering the unpredictability of the clinical course, it became clear that there is a need for good severity assessment tools for patients with COVID-19 seeking medical care for the first time.

Clinical manifestations of COVID-19 were directly dependent on immune system activation. If there is a dysregulated immune response to infection, there could be excessive damage to organs and tissues and the development of severe disease presentation with organ failure, which can quickly lead to death [4]. Besides excessive inflammation, factors such as coagulation abnormalities, endothelial dysfunction, and infiltration of organs by inflammatory cells can lead to multiorgan failure [5]. Acute-phase reactants (APRs) are groups of various proteins whose concentration changes as a response to cytokines synthesized by local tissue-dwelling cells as a response to tissue injury, infection, or other inflammatory processes. APRs can be classified into two groupsbased on their behavior during acute-phase response: positive APRs whose concentration rises (e.g., serum amyloid A [SAA], C-reactive protein [CRP], procalcitonin [PCT], interleukin-6 [IL-6], ferritin, fibrinogen, and D-dimer) and negative APRs which concentration gets lower (e.g., albumin, transferrin). Because APR levels can be perceived as an indirect measure of immune system activation, determining their levels became a significant part of daily practice for different inflammatory and infectious diseases [6]. During COVID-19, there is an innate and adaptive immune system activation, which, if pronounced, leads to clinical manifestations of the infection. The amount and speed of changes in the production of various APRs depend on the strength of the immune response, with stronger responses leading to faster and more significant changes [7]. Classification of APRs can also be based on the magnitude of change in their concentration:major APRs have a 10-100-fold or more increase (e.g., SAA and CRP), moderate 2-10-fold increase (e.g., α_1_-acid glycoprotein), and those with less than a 2-fold increase are considered minor (e.g., fibrinogen) [8]. The appeal of SAA in investigating various inflammatory and infectious diseases, including COVID-19, is due to its ability to rapidly increase its concentration by more than 1000 times during the first 24-36 hours after the onset of tissue injury and subsequent inflammation. As a result, the serum levels of SAA are closely linked to the severity of the disease process. SAA represents a group of several very similar proteins that are synthesized in minimal amounts in the absence of inflammation. In humans, based on the amino acid sequence, SAA can be divided into SAA1, SAA2, and SAA4 [9]. During the acute-phase reaction, the serum concentrations of SAA1 and SAA2 rise quickly due to accelerated transcription of their respective genes [10] as a response to proinflammatory cytokines such as tumor necrosis factor-α (TNF-α), interleukins (e.g., 1α, 1β, and 6), transforming growth factor-β (TGF-β), and interferon-y (INF-y) [9]. So far, many studies have evaluated APRs to assess disease severity and prognosis of SARS-CoV-2 infection [7] [11] [12] [13] [14] [15] [16] [17] [18] [19], mostly in hospitalized patients, where SAA showed a positive correlation with disease severity and prognosis. It also demonstrated better characteristics for the same purpose when compared to other APRs [13] [20] [21] [22].

This study aimed to assess the usefulness of SAA and other APRs in the cohort of ambulatory care patients with COVID-19 to identify possible relationships between these APRs and disease outcomes (disease severity, hospital admission, and survival).

## Materials and methods

### Study setting and participants

We conducted the prospective cohort study in the outpatient setting of the Clinic for Infectious and Tropical Diseases »Prof. Dr. Kosta Todorovi}«, University Clinical Center of Serbia, from August to November 2020. In this period, institution was a part of the so-called COVID system as a triage and treatment center, which exclusively managed patients with suspected or proven SARS-CoV-2 infection. After initial evaluation in the primary care setting, patients would be referred, at the discretion of a general practitioner, to our outpatient department for further evaluation. Based on this evaluation, we would then decide if a patient needed further inpatient treatment or if it could be managed as an outpatient. The study included only adult patients (age ≥ 18 years), irrespective of gender, with symptomatic SARS-CoV-2 infection lasting less than 14 days, confirmed by realtime polymerase chain reaction (RT-PCR) in nasopharyngeal swab samples. The last inclusion criterion was the absence of other co-infections at the moment of evaluation.

During an evaluation, demographic parameters such as age, gender, and additional information onpresent chronic illnesses (hypertension, cardiovascular diseases [angina, previous myocardial infarction, heart failure, arrythmia], asthma, chronic kidney disease, active malignancy, chronic liver disease, and immunosuppression) were documented. Information was also gathered on the presence or absence of 21 potential symptoms of COVID-19 infection, such as fever, chills, malaise, easy fatiguability, loss of appetite, cough, nasal discharge, sneezing, sore throat, chest pain, chest pressure, shortness of breath, anosmia, ageusia, headache, back pain, myalgia/arthralgia, diarrhea, nausea, vomiting, and abdominal pain. The length of SARS-CoV-2 infection before seeking care at our institution was also documented as part of the data collection. After history taking, a detailed physical examination with vital signs assessment (body temperature, pulse rate, blood pressure, respiratory rate), measurement of peripheral oxygen saturation by pulse oximetry, and anthropomorphic measurements (body height and weight) were performed. Body mass index (BMI) was calculated by dividing weight (kg) by height squared (m^2^), and the patients were classified into two groups (normal weight and overweight [BMI > 30 kg/m^2^]). After that, the peripheral venous blood samples were collected. For all blood samples, we determined positive APRs, including SAA, CRP, IL-6, PCT, ferritin, fibrinogen, and D-dimer, and negative APRs, such as albumin and transferrin. The diagnosis of pneumonia was made based on the combination of lung auscultation and chest X-ray imaging findings. Based on the collected data, patients were classified into severity groups (mild, moderate, and severe disease) by criteria established by WHO [23]. Then, by the clinical judgment of the examining physician, patients would be hospitalized if they accepted inpatient treatment, or they would be sent home if they refused hospitalization or if hospitalization was deemed unnecessary. The disease outcomes were assessed immediately at the end of inpatient treatment for hospitalized patients and one month after the first encounter for outpatients by insight into the Belgrade City Institute for Public Health’s database of deceased persons.

This study was approved by the Ethics Commission of the Faculty of Medicine, University of Belgrade, under the number 1322/II-1, 2. We acquired written consent from all the patients after a detailed explanation of the study.

### Determination of acute-phase reactants

APRs were examined in blood samples. Blood samples were obtained by antecubital vein punctureusing standard operating procedures during collection [24]. Closed systems for venipuncture (Becton & Dickinson Vacutainer Systems, Franklin Lakes, New Jersey) were used to obtain blood samples (vacutainers, 22 Standard Wire Gauge [SWG], and reusable adapters). Serum samples were obtained using vacutainers with clot activator (BD Vacutainer® SST™ Tubes). Plasma samples were obtained using citrate as an anticoagulant (BD Vacutainer® Plastic citrate tube, Buffered sodium citrate [0.109 M, 3.2%]). Excluding the measurement of SAA, all analyses were done in the clinical laboratories of the University Clinical Center of Serbia immediately after sampling. CRP and albumin were determined on Dimension RxL Max (Siemens Healthcare GmbH). Ferritin and transferrin were measured in serum using an immunoturbidimetry method on the Roche Cobas c502 immunochemistry analyzer. In comparison, PCT and IL-6 were quantified by electrochemiluminescence on Roche Cobas e602 automated immunochemistry analyzer. Fibrinogen concentrations were measured by photo-optic coagulometry, and D-dimer concentrations by immunoturbidimetry on Sysmex CA1500 automated hemostasis analyzer (Sysmex Europe GmbH). For the determination of SAA, after aliquoting, the samples were stored at -20°C until analysis. SAA was determined in serum samples at the Institute of Medical Biochemistry of the Military Medical Academy, Belgrade, Serbia, by a commercial N Latex SAA Assay on a BNII™ System (Siemens Healthcare GmbH) immunochemical nephelometric analyzer. The reference values for SAA, CRP, IL-6, PCT, ferritin, fibrinogen, D-dimer, transferrin, and albumin were 6.4 mg/L, 10.0 mg/L, 7 ng/L, 0.1 μg/L, 30–400 μg/L (male) and 13–150 μg/L (female), 1.8–3.5 g/L, 0.5 mg/L FEU (fibrinogen equivalent units), 2.0–3.6 g/L, 34–55 g/L, respectively. The operating procedures are closely followed, including all the parameter settings and experimental steps.

### Statistical analysis

IBM SPSS statistical software version 26 (SPSS 26 Chicago, Illinois, USA) was used to analyze study data. Data were described as counts (n) and percentages (%). The numerical data were expressed as means and standard deviations (SD) if normally distributed and as median/interquartile range for non-normal distribution. To assess one-group proportion, we used a binomial test. For the data distribution analysis, the Kolmogorov-Smirnov test was used. Two and three independent samples with normally distributed data were analyzed with a t-test for independent samples and ANOVA, respectively. For non-normally distributed data, we used the Mann-Whitney U test for two samples and the Jonckheere-Terpstra (J-T) test for three ordered samples. A post hoc pairwise comparison between groups was performed if the ANOVA or J-T test result was statistically significant. Categorical variables were analyzed with a Chi-square test and Fisher’s exact test. We used exact p-values obtained through non-parametric tests and contingency table analysis whenever it was computationally feasible without exceeding the computer’s memory capacity.

On the other hand, when such a calculation was impossible, we estimated p-values using the Monte Carlo method. In this method, we set a confidence interval of 99% and generated 1,000,000 samples to estimate the p-value. Correlations between variables were explored by the non-parametric Spearman’s rho (*p*).

The APR levels were compared between disease severity groups, hospitalized and non-hospitalized patients, and survivors and non-survivors. For SAA, CRP, IL-6, PCT, fibrinogen, D-dimer, transferrin, and albumin, we used values irrespective of gender. For group comparison using ferritin levels, genders were analyzed separately, considering different, gender-specific reference values. Receiver-operating characteristic (ROC) curve analysis was used to determine the Area-under-the-curve (AUC) of APRs used in the study. For optimal cut-off values and their corresponding sensitivity and specificity determination, we used the Youden index.

A *p*-value < 0.05 was considered statistically significant, and a *p*-value < 0.01 was consideredhighly statistically significant (two-sided tests). For multiple tests, *p*-values were adjusted by Bonferroni correction.

## Results

### Patient characteristics

The baseline patient characteristics have been summarized in Table 1. The study included 192 patients, 82 (42.7%) women and 110 (57.3%) men, with an average age of 53.0 (15.9) years, and arange of 19–94 years. At least one comorbidity was present in 118 (61.5%) patients, and the most common was arterial hypertension which was present in 74 (38.5%) patients. Comorbidities were significantly more common in hospitalized than non-hospitalized patients (x^2^ [1] = 6.428, p = 0.012). However, there was no difference between disease severity groups (x^2^ [2] = 4.410, p = 0.112) and between survivors and non-survivors (x^2^ [1] = 4.273, p = 0.053) in the presence of comorbidities.

**Table 1:** Demographics and baseline characteristics of patients with COVID-19. SD, standard deviation; IQR, interquartile range<br>^*^ Based on 2-test statistical analysis, except for age (binomial test), gender (t-test for independent samples), and duration of illness (Jonckheere-Terpstra test).<br>† Ischemic heart disease, cardiomyopathy, atrial fibrillation, heart failure.

Variable	All<br>N (%)	Male<br>N (%)	Female<br>N (%)	*p*-value^*^
Gender	192 (100.0)	110 (57.3)	82 (42.7)	0.051
Age - years (SD)	53.0 (15.9)	54.8 (15.7)	50.6 (15.9)	0.066
Comorbidities (5 most common)				
Hypertension	74 (38.5)	47 (42.7)	27 (32.9)	0.180
Cardiovascular disease†	32 (16.7)	19 (17.3)	13 (15.9)	0.847
Obesity ( 30 kg/m^2^)	30 (15.6)	20 (18.2)	10 (12.2)	0.317
Diabetes mellitus	19 (9.9)	12 (10.9)	7 (8.5)	0.634
Asthma	9 (4.7)	2 (1.8)	7 (8.5)	0.039
Clinical characteristics of COVID-19				
Fever	174 (90.6)	101 (91.8)	73 (89.0)	0.618
Malaise	161 (83.9)	89 (80.9)	72 (87.8)	0.237
Cough	132 (68.8)	79 (71.8)	53 (64.6)	0.345
Myalgia/arthralgia	106 (55.2)	50 (45.5)	56 (68.3)	0.002
Easy fatiguability	104 (54.2)	59 (53.6)	45 (54.9)	0.885
Loss of appetite	83 (43.2)	47 (42.7)	36 (43.9)	0.884
Headache	75 (39.1)	30 (27.3)	45 (54.9)	<0.001
Chills	60 (31.3)	32 (29.1)	28 (34.1)	0.529
Diarrhea	58 (30.2)	32 (29.1)	26 (31.7)	0.752
Sore throat	50 (26.0)	22 (20.0)	28 (34.1)	0.031
Anosmia	49 (25.5)	24 (21.8)	25 (30.5)	0.184
Back pain	46 (24.0)	23 (20.9)	23 (28.0)	0.306
Nasal discharge	45 (23.4)	18 (16.4)	27 (32.9)	0.010
Shortness of breath	45 (23.4)	24 (21.8)	21 (25.6)	0.606
Nausea	45 (23.4)	20 (18.2)	25 (30.5)	0.058
Ageusia	44 (22.9)	22 (20.0)	22 (26.8)	0.300
Chest pressure	43 (22.4)	23 (20.9)	20 (24.4)	0.602
Chest pain	33 (17.2)	18 (16.4)	15 (18.3)	0.847
Sneezing	20 (10.4)	8 (7.3)	12 (14.6)	0.150
Vomiting	16 (8.3)	9 (8.2)	7 (8.5)	1.000
Abdominal pain	16 (8.3)	10 (9.1)	6 (7.3)	0.794
Duration of illness in days- median (IQR)	6.0 (4.0–8.0)	6.0 (4.0–8.0)	6.0 (4.0–8.0)	0.507
COVID-19 severity				
Mild	42 (21.9)	17 (15.5)	25 (30.5)	0.042
Moderate	113 (58.9)	71 (64.5)	42 (51.2)	
Severe	37 (19.3)	22 (20.0)	15 (18.3)	
Hospitalization				
No	92 (47.9)	46 (41.8)	46 (56.1)	0.058
Yes	100 (52.1)	64 (58.2)	36 (43.9)	
COVID-19 outcome after one month				
Survived	181 (94.3)	100 (90.9)	1 (1.2)	0.026
Died	11 (5.7)	10 (9.1)	81 (98.8)	

Fever was the most frequent symptom (174 [90.6%] patients), while malaise and cough were the next most frequent (161 [83.9%] and 132 [68.8%], respectively). With increasing disease severity from mild over moderate to severe, there was a trend for an increase in the median number of symptoms (6 [4–9] vs. 7 [4–10] vs. 9 [7–11], T_JT_ = 6.228, z = 2.525, p = 0.011). Hospitalized patients had more symptoms than outpatients (8 [4–9] vs. 6 [5–11], *U* = 3640.0, p = 0.012). The median disease duration before patients visited our institution was 6.0 (4–8) days. Most patients had moderate disease (113 [58.9%]). Mean age was significantly different between the mild, moderate, and severe disease groups (40.2 [12.6] vs. 54.8 [15.1] vs. 62.2 [12.6] years, *F* [2, 189] = 26.070, *p* < 0.001), between hospitalized and non-hospitalized patients (44.6 [13.2] vs. 60.8 [14.1] years, *t* [190] = -8.201, p < 0.001), and between survivors and non-survivors (51.7 [15.1] vs. 75.1 [11.3] years, t [190] = 5.048, *p* < 0.001). After clinical and diagnostic evaluation, 100 (52.1%) patients were hospitalized, and the male gender was predominant (64 [64.0%], *p* = 0.006). Out of 192 patients, 5 (11.9%) with mild, 60 (53.1%) with moderate, and 35 (94.6%) patients with severe disease were hospitalized.

There were 11 (5.7%) patients who had a fatal outcome. The mean age of non-survivors was significantly higher when compared to survivors (75.1 [11.3] vs. 51.7 [15.1] years, *t* [190] = 5.048, *p* < 0.001). There were no fatal outcomes in the group of non-hospitalized patients, so the mortality in a group of hospitalized patients was 11%. Most patients who died were male (10 [90.9%], *p* < 0.001). The mortality in the mild, moderate, and severe disease groups was 2.4%, 3.5%, and 16.2%, respectively, and the difference between groups was statistically significant (x^2^ = 7.270, *p* = 0.017). Post-hoc analysis showed that mortality was significantly higher in the severe disease group than in the moderate disease group (*p* = 0.015). There was no difference in mortality between the mild and moderate and mild and severe disease groups.

### Serum amyloid A

SAA was elevated (≥ 6.4 mg/L) in 148 (77.1%) patients. Patients with normal SAA values were significantly younger compared to those who had abnormal values (40.8±13.2 years vs. 56.6±14.8 years, t [190] = −6.375, p < 0.001). Also, the normal SAA group had a higher proportion of female patients (68.2% vs. 35.1%), fewer patients with comorbidities (40.9% vs. 67.6%), and tended to have more patients with mild disease (56.8% vs. 11.5%). There was no difference between groups of patients with normal and abnormal SAA values in the frequency of fatal outcomes (2.3% vs. 6.8%, p = 0.462). Patients with comorbidities had higher median SAA levels than those without (76.1 [11.5–233.6] vs. 15.1 [3.6–80.3], *U* = 3045.0, p < 0.001). There was a trend of higher median SAA levels from mild over moderate to severe disease group (T_JT_ = 8442.0, *z* = 8.176, *p* < 0.001). Post-hoc pairwise comparisons indicated that the median SAA values significantly differed between mild and moderate, mild and severe, and moderate and severe groups (*p* < 0.001 for all three comparisons). The SAA levels were significantly higher in patients who were hospitalized and who did not survive (*p* < 0.001, and *p* < 0.001, respectively) (Table 2).

**Table 2:** Difference between disease severity groups, hospitalized and non-hospitalized, and survivors and non-survivors in median values of acute-phase reactants. M, male; F, female; IQR, interquartile range; APRs, acute–phase reactants; SAA, serum amyloid A; CRP, C–reactive protein; IL–6, interleukin–6; PCT, procalcitonin.<br>* Based on Jonckheere–Terpstra test statistical analysis.<br>† Based on Mann–Withney U test statistical analysis.<br>‡ Because of gender–specific reference values, descriptive statistics for the whole sample were not calculated.<br>§ Only one female patient died; therefore, measured values were given instead of the median; p–value should be interpreted with caution due to the small sample size.

Parameter		All	COVID–19 severity			*p*-value^†^	Hospitalization		*p*-value^†^	COVID–19 outcome		*p*-value^†^
			Mild<br>median (IQR)	Moderate<br>median (IQR)	Severe<br>median (IQR)		No<br>median (IQR)	Yes<br>median (IQR)		Survived<br>median (IQR)	Died<br>median (IQR)	
Positive APRs												
SAA<br>(mg/L)	All	32.5<br>(5.8–218.0)	4.1<br>(3.5–11.6)	42.3<br>(11.0–202.0)	219.0<br>(70.4–557.5)	<0.001	8.8<br>(3.5–22.9)	143.5<br>(49.8–497.3)	<0.001	30.1<br>(7.3–175.3)	231.0<br>(98.7–503.0)	0.008
	M	95.5<br>(13.2–335.7)	7.6<br>(3.5–12.7)	102.3<br>(18.7–342.0)	303.5<br>(143.2–985.6)	<0.001	12.7<br>(4.8–88.3)	219.0<br>(75.3–607.5)	<0.001	78.5<br>(11.5–325.8)	250.5<br>(94.2–538.5)	0.117
	F	15.7<br>(3.5–66.0)	3.5<br>(3.5–9.7)	17.1<br>(5.0–61.1)	96.2<br>(48.9–421.0)	<0.001	5.0<br>(3.5–16.4)	66.2<br>(19.1–172.0)	<0.001	15.5<br>(3.5–57.7)	134.0§<br>–	0.134
CRP<br>(mg/L)	All	21.7<br>(3.4–55.8)	2.9<br>(1.6–7.3)	25.1<br>(4.3–50.4)	86.1<br>(33.0–125.9)	<0.001	3.9<br>(1.9–17.3)	47.1<br>(24.0–100.8)	<0.001	17.4<br>(3.4–50.5)	72.4<br>(43.9–143.8)	0.002
	M	33.5<br>(13.1–88.1)	3.5<br>(2.1–14.0)	32.6<br>(14.5–76.6)	91.7<br>(60.6–146.1)	<0.001	14.4<br>(2.9–29.1)	72.2<br>(31.3–129.9)	<0.001	31.3<br>(11.4–81.9)	80.2<br>(40.5–150.1)	0.050
	F	6.2<br>(1.9–24.1)	2.3<br>(1.2–6.0)	6.3<br>(3.0–24.1)	38.1<br>(11.0–101.0)	<0.001	2.9<br>(1.7–7.2)	24.3<br>(6.6–47.4)	<0.001	6.1<br>(1.9–23.5)	56.6§<br>–	0.171
IL–6<br>(ng/L)	All	19.9<br>(4.7–43.7)	2.5<br>(1.4–5.4)	21.6<br>(8.8–40.5)	50.9<br>(28.2–68.7)	<0.001	5.3<br>(2.0–16.2)	37.9<br>(20.4–56.4)	<0.001	18.2<br>(4.0–40.5)	63.4<br>(38.6–125.4)	<0.001
	M	27.9<br>(10.5–51.1)	2.5<br>(1.5–7.8)	27.5<br>(12.2–44.1)	54.1<br>(43.2–76.6)	<0.001	9.5<br>(2.5–39.3)	44.1<br>(28.0–64.8)	<0.001	24.4<br>(9.0–45.6)	67.8<br>(47.6–126.2)	<0.001
	F	8.8<br>(3.0–26.6)	2.7<br>(1.4–5.6)	11.0<br>(4.8–23.9)	28.6<br>(20.4–55.1)	<0.001	3.9<br>(1.9–9.4)	25.2<br>(10.9–40.9)	<0.001	8.5<br>(3.0–57.0)	38.6§<br>–	0.341
PCT<br>(μg/L)	All	0.07<br>(0.04–0.10)	0.04<br>(0.03–0.06)	0.07<br>(0.05–0.10)	0.1<br>(0.06–0.14)	<0.001	0.05<br>(0.03–0.07)	0.09<br>(0.06–0.14)	<0.001	0.06<br>(0.04–0.09)	0.17<br>(0.13–0.22)	<0.001
	M	0.09<br>(0.06–0.13)	0.05<br>(0.04–0.06)	0.09<br>(0.06–0.13)	0.13<br>(0.08–0.16)	<0.001	0.06<br>(0.05–0.09)	0.11<br>(0.07–0.18)	<0.001	0.08<br>(0.05–0.12)	0.16<br>(0.12–0.22)	0.002
	F	0.05<br>(0.03–0.07)	0.04<br>(0.02–0.05)	0.05<br>(0.04–0.07)	0.07<br>(0.05–0.09)	0.002	0.04<br>(0.02–0.05)	0.05<br>(0.05–0.09)	<0.001	0.05<br>(0.03–0.07)	0.19§<br>–	0.024
Ferritin^‡^(μg/L)	M	612.9<br>(368.9–	359.5<br>(216.2–421.2)	609.3<br>(394.3–980.1)	879.8<br>(647.9–1697.2)	<0.001	435.7<br>(298.3–622.5)	742.4<br>(538.8–1287.1)	<0.001	589.7<br>(367.3–905.5)	1085.5<br>(451.0–1809.1)	0.131
	F	149.8<br>(54.4–282.3)	110.7<br>(41.3–217.4)	149.0<br>(54.4–279.0)	261.4<br>(172.2–639.7)	0.005	124.9<br>(46.1–190.0)	212.6<br>(118.9–430.1)	0.001	146.1<br>(54.4–283.6)	274.2§<br>–	0.561
Fibrinogen<br>(g/L)	All	4.0<br>(3.1–5.1)	3.0<br>(2.6–4.0)	4.1<br>(3.5–5.1)	5.0<br>(3.7–8.1)	<0.001	3.6<br>(3.0–4.5)	4.5<br>(3.5–5.7)	<0.001	4.0<br>(3.1–5.1)	4.1<br>(3.2–4.9)	0.850
	M	4.2<br>(3.3–5.7)	3.0<br>(2.6–4.3)	4.3<br>(3.5–5.2)	5.9<br>(3.9–8.1)	0.001	3.9<br>(3.0–5.0)	4.6<br>(3.7–6.5)	0.005	4.3<br>(3.3–5.7)	4.1<br>(3.6–5.1)	0.811
	F	3.7<br>(3.0–4.7)	3.0<br>(2.6–3.9)	3.6<br>(3.2–4.6)	4.8<br>(3.4–6.0)	0.001	3.3<br>(2.9–4.1)	4.2<br>(3.1–5.4)	0.011	3.7<br>(3.0–4.7)	3.1§<br>–	0.671
D-dimer<br>(mg/L<br>FEU)	All	0.52<br>(0.30–0.97)	0.41<br>(0.20–0.56)	0.55<br>(0.27–0.99)	0.57<br>(0.41–1.01)	0.009	0.45<br>(0.25–0.85)	0.61<br>(0.34–1.06)	0.004	0.52<br>(0.30–0.96)	0.71<br>(0.39–2.18)	0.058
	M	0.54<br>(0.31–1.01)	0.30<br>(0.19–0.43)	0.61<br>(0.34–1.11)	0.61<br>(0.46–1.20)	0.001	0.50<br>(0.28–0.85)	0.61<br>(0.33–1.27)	0.005	0.53<br>(0.31–0.99)	0.69<br>(0.36–1.96)	0.196
	F	0.50<br>(0.29–1.75)	0.49<br>(0.30–1.12)	0.51<br>(0.23–0.90)	0.53<br>(0.35–0.95)	0.669	0.42<br>(0.24–0.86)	0.59<br>(0.37–0.97)	0.055	0.49<br>(0.28–1.67)	11.33§	0.012
Negative APRs												
Transferrin<br>(g/L)	All	2.16<br>(1.92–2.41)	2.23<br>(2.05–2.64)	2.17<br>(1.91–2.41)	2.01<br>(1.74–2.23)	0.001	2.22<br>(2.05–2.63)	2.02<br>(1.80–2.23)	<0.001	2.17<br>(1.95–2.42)	1.75<br>(1.61–2.10)	0.006
	M	2.10<br>(1.83–2.32)	2.25<br>(2.18–2.59)	2.09<br>(1.83–2.26)	2.01<br>(1.63–2.10)	0.001	2.20<br>(1.98–2.43)	2.00<br>(1.75–2.21)	0.001	2.12<br>(1.88–2.36)	1.80<br>(1.80–2.26)	0.048
	F	2.22<br>(2.05–2.64)	2.16<br>(2.02–2.73)	2.30<br>(2.13–2.69)	2.12<br>(1.91–2.33)	0.252	2.33<br>(2.13–2.77)	2.17<br>(1.91–2.35)	0.003	2.23<br>(2.06–2.66)	1.55§	0.024
Albumin<br>(g/L)	All	39.0<br>(36.0–42.0)	42.0<br>(39.0–44.0)	39.0<br>(36.0–41.5)	36.0<br>(34.0–37.0)	<0.001	41.0<br>(38.0–43.0)	36.5<br>(34.0–39.7)	<0.001	39.0<br>(36.0–42.0)	35.0<br>(30.0–37.0)	<0.001
	M	38.0<br>(35.0–41.3)	43.0<br>(41.0–44.0)	38.0<br>(34.0–41.0)	35.0<br>(33.5–37.0)	<0.001	41.0<br>(37.8–43.0)	36.0<br>(33.2–40.0)	<0.001	39.0<br>(35.0–42.0)	35.5<br>(30.8–37.0)	0.006
	F	39.0<br>(36.0–42.0)	42.0<br>(38.0–44.0)	39.5<br>(37.0–42.0)	36.0<br>(35.0–37.0)	<0.001	41.5<br>(38.8–43.3)	37.0<br>(35.0–39.0)	<0.001	39.0<br>(36.5–42.0)	22§	0.024

**Figure 1:**
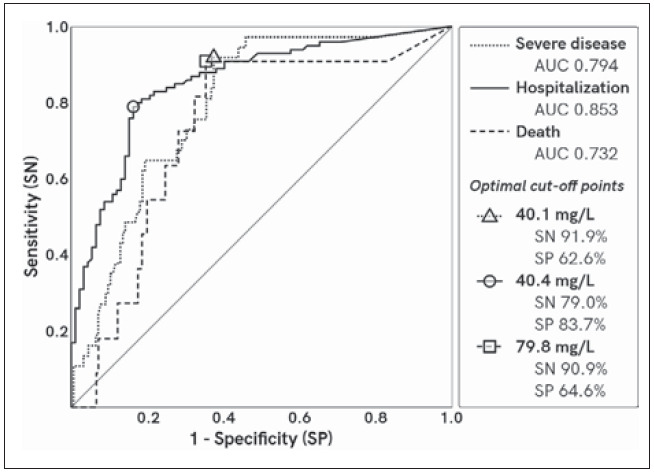


ROC curves for SAA are shown in Figure 1. Based on ROC curve analysis AUC for SAA has fair classification performance for disease severity (0.794) and death (0.732) and good performancefor hospitalization (0.853). Optimal SAA cut-off values for severe disease, hospitalization, and death were 44.1 mg/L (sensitivity 91.9%, specificity 62.6%), 40.4 mg/L (sensitivity 79.0%, specificity 83.7%), 79.8 mg/L (sensitivity 90.9%, specificity 72.9%), respectively. Detailed results of ROC curve analyses are shown in Table 3-Table 4.

**Table 3:** Acute-phase reactants ROC curves analysis of for COVID-19 severity (non–severe = mild and moderate vs. severe disease). M, male; F, female; AUC, area-under-the-curve; CI, confidence interval; SN, sensitivity; SP, specificity; APRs, acute-phase reactants; SAA, serum amyloid A; CRP, C-reactive protein; IL-6, interleukin-6; PCT, procalcitonin.<br>^*^ Optimal cut-off, sensitivity, and specificity were not calculated for AUC with p > 0.05.

Parameter	AUC	95% CI	*p*-value	Optimal^*^cut-off	SN (%)	SP (%)
Positive APRs						
SAA (mg/L)	0.794	0.723–0.865	<0.001	>40.1	91.9	62.6
CRP (mg/L	0.789	0.709–0.869	<0.001	>38.0	75.7	76.1
IL–6 (ng/L)	0.800	0.729–0.871	<0.001	>22.60	86.5	63.9
Procalcitonin (mg/L)	0.685	0.594–0.777	<0.001	>0.07	75.7	56.5
Ferritin (μg/L) M<br>F	0.737	0.620–0.853	0.001	>637.0	81.8	63.6
	0.731	0.591–0.871	0.005	>163.4	86.7	62.7
Fibrinogen (g/L)	0.704	0.607–0.801	<0.012	>4.7	64.9	72.3
D–dimer (mg/L FEU)	0.585	0.498–0.673	0.108	–		
Negative APRs						
Transferrin (g/L)	0.661	0.563–0.759	0.002	2.13	70.3	61.3
Albumin (g/L)	0.769	0.694–0.843	<0.001	37.5	83.8	69.0

**Table 4:** Acute-phase reactants ROC curves analysis of for assessment outcome (hospitalization). M, male; F, female; AUC, area-under-the-curve; CI, confidence interval; SN, sensitivity; SP, specificity; APRs, acute-phase reactants; SAA, serum amyloid A; CRP, C-reactive protein; IL-6, interleukin-6; PCT, procalcitonin.

Parameter	AUC	95% CI	p-value	Optimal<br>cut-off	SN (%)	SP (%)
Positive APRs						
SAA (mg/L)	0.853	0.798–0.907	<0.001	>40.4	79.0	83.7
CRP (mg/L)	0.830	0.772–0.887	<0.001	>21.8	77.0	79.3
IL-6 (ng/L)	0.860	0.807–0.913	<0.001	>19.5	79.0	80.4
Procalcitonin (mg/L)	0.774	0.709–0.839	<0.001	>0.07	71.0	68.5
Ferritin (μg/L) M<br>F	0.747	0.654–0.839	<0.001	>537.0	76.6	65.2
	0.714	0.601–0.828	0.001	>155.9	72.2	71.7
Fibrinogen (g/L)	0.673	0.597–0.748	<0.001	>4.1	63.0	65.2
D-dimer (mg/L FEU)	0.619	0.540–0.698	0.005	>0.59	54.0	68.5
Negative APRs						
Transferrin (g/L)	0.698	0.625–0.771	<0.001	≤2.03	50.0	80.4
Albumin (g/L)	0.767	0.700–0.834	<0.001	≤38.5	68.0	72.8

### Other acute-phase reactants

CRP, IL-6, fibrinogen, D-dimer, and ferritin levels were elevated in 115 (59.9%), 131 (68.2%), 120 (62.5%), 101 (52.6%), and 119 (62.0%) patients, respectively, while PCT levels were above the reference value of 0.1 μg/L in 56 (29.2%) and even in fewer patients (11, 5.7%) above the standard cut-off value for marked infection of 0.25 μg/L. Albumin and transferrin levels were depressed in 22 (11.5%) and 58 (30.2%) patients. CRP, IL-6, PCT, ferritin (male and female), and D-dimer median levels were significantly higher (*p* = 0.002, *p *< 0.001, *p* < 0.001, *p *= 0.019, *p *= 0.010, and *p* = 0.041, respectively), and albumin levels were significantly lower (*p *< 0.001) in the patients with comorbidities. In contrast, median fibrinogen and transferrin levels were not significantly different between patients with and without comorbidities (*p* = 0.509, *p* = 0.061, respectively). There was a significant difference in all APR median values between severity groups and hospitalized andnon-hospitalized patients, but only for CRP, IL-6, PCT, transferrin, and albumin between survivors and nonsurvivors (Table 2). The ROC curve analysis showed that only AUC for IL-6 had good classification performancefor severe disease (0.800), while CRP, ferritin for both males and females, fibrinogen and albumin had fair performance (0.789, 0.737, 0.731, 0.704, and 0.769, respectively). PCT and transferrin had poor performance (0.685 and 0.661, respectively), and D-dimer failed to show any classification performance (0.585, *p* = 0.108) (Table 3).

For hospitalization, only AUC for CRP and IL-6 showed good classification performance (0.830 and 0.860, respectively), while AUC for PCT, ferritin for both males and females, and albumin showed fair (0.774, 0.747, 0.714, and 0.767, respectively) and for fibrinogen, D-dimer and transferrin poor performance (0.671, 0.619, and 0.698, respectively) (Table 4).

Classification performance for death, based on AUC, was good for IL-6, PCT, and albumin (0.865, 0.858, and 0.813, respectively) The fair performance had AUC for CRP and transferrin (0.769 and 0.769, respectively). AUC for ferritin in male patients, fibrinogen, and D-dimer failed to show any classification performance (0.646, *p* = 0.129, 0.517, *p* = 0.847, and 0.670, *p* = 0.058, respectively) (Table 5).

**Table 5:** Acute-phase reactants ROC curves analysis of for COVID-19 outcome (death). M, male; AUC, area-under-the-curve; CI, confidence interval; SN, sensitivity; SP, specificity; APRs, acute-phase reactants; SAA, serum amyloid A; CRP, C-reactive protein; IL-6, interleukin-6; PCT, procalcitonin.<br>^*^ Optimal cut-off, sensitivity, and specificity were not calculated for AUC with *p* > 0.05.<br>^†^ Only one female patient died; therefore, no ROC curve analysis can be performed.

Parameter	AUC	95% CI	p-value	Optimal^*^<br>cut-off	SN (%)	SP (%)
Positive APRs						
SAA (mg/L)	0.732	0.592–0.872	0.01	>79.8	90.9	64.6
CRP (mg/L)	0.769	0.640–0.899	0.003	>42.1	81.8	71.8
IL-6 (ng/L)	0.865	0.788–0.941	<0.001	>36.8	90.9	72.9
Procalcitonin (mg/L)	0.858	0.748–0.968	<0.001	>0.13	81.8	86.7
Ferritin (mg/L) ^†^ M	0.646	0.425–0.867	0.129	–		
Fibrinogen (g/L)	0.517	0.373–0.661	0.847	–		
D-dimer (mg/L FEU)	0.670	0.469–0.845	0.058	–		
Negative APRs						
Transferrin (g/L)	0.769	0.637–0.901	0.003	1.86	63.6	83.4
Albumin (g/L)	0.813	0.715–0.912	<0.001	37.5	90.9	61.9

### Correlation between SAA and other APRs

There was a strong correlation between SAA and CRP values (*p* = 0.862,* p* < 0.001) and SAA and IL-6 values (*p* = 0.781, *p* < 0.001), moderate correlation between SAA and albumin (*p* = -0.647, *p* < 0.001), PCT (*p* = 0.634, *p* < 0.001), fibrinogen (*p* = 0.647, *p* < 0.001), and transferrin (*p* = -0.634,*p* < 0.001), but only weak correlation with D-dimer (*p* = 0.342, *p* < 0.001).

## Discussion

After three years of the SARS-CoV-2 pandemic, infection with this virus still presents a global challenge, especially because new viral variants are expected. There is an unpredictability of the infection course in different patients and a possibility of developing the severe disease in a matter of days and hours. Therefore, it is of utmost importance to have readily available clinical tools for assessing disease severity with good sensitivity and specificity for the unfavorable course, whether it is the need for hospitalization or a potentially fatal outcome. The measurement of APR levels can help deliver appropriate guidance in clinical management. This study examines the usefulness of SAA as a relatively rarely used APR and other more commonly used laboratory parameters in assessing COVID-19 in ambulatory care patients.

When used in COVID-19 patients, the measurement of SAA levels in our study proved useful in disease assessment, which is consistent with previous findings [13] [20] [21] [25]. Our finding showed that the SAA outperformed many other APRs for detecting patients with severe illness and those who need hospitalization but not for predicting death from COVID-19, which has a lower but still significant value. Cheng et al. [13] showed that periodic measurements of SAA during hospitalization based on ROC curve analysis are suitable for predicting death and that the value of SAA measurement has the lowest sensitivity (80.6%) and AUC (0.588) on day 1 of hospitalization, which is markedly lower than in our study. However, their study included only hospitalized patients with severe and critical forms of COVID-19. Also, they compared SAA with CRP and found no difference in predictive value. In the study of Li et al. [20], the results of the ROC curve analysis showed that SAA’s ability to predict disease severity, as measured by the AUC, was 0.718. This value was lower than what was found in our study. This difference can be attributed to the smaller number of patients in their study and the unequal distribution of patients across different levels of disease severity. Another finding from Li et al. study, which deserves to be mentioned, is that SAA levels on admission correlate well with CT lung findings and progression of lung involvement on repeated imaging, even after a few days. In their study, Chen et al. [21] showed that CRP has abetter predictive value for developing the severe disease (ARDS) than SAA, which is opposite to our study findings. However, this can be explained partially by the fact that their study included only hospitalized patients who were seriously ill. In some studies, combining SAA with other APRs improved diagnostic and prognostic utility. For example, Chen et al. [21] combined SAA, CRP, and white blood cell count (WBC) and got improvement in the AUC of SAA as an individual marker for the detection of severe disease (ARDS) from 0.712 to 0.878. Conversely, Liu et al. [22] showed that a combination of SAA with IL-6 can improve the AUC of SAA for severe disease from 0.865 to 0.904. When a patient presents with SARS-CoV-2 infection in an outpatient setting, based on these findings, physicians should remember that patients with elevated SAA levels may have an increased risk of becoming severely ill.

When compared to SAA for assessment of viral infections, Miwata et al. [26] showed that the CRP levels are not a reliable indicator of viral diseases because, in more than half the cases, it was normal while SAA levels are often elevated. Our findings were similar, considering that more than one-third of our patients had normal CRP levels. On the other hand, only approximately one-fifth had normal SAA levels, confirming SAA as more sensitive for detecting even low-level inflammation. Our study results showed that CRP is a useful marker during COVID-19, both for illness severity assessment and disease prognosis, consistent with previous studies [13] [25] [27]. However, in our study and studies of Wang et al. [25] and Cheng et al. [13], SAA has better diagnostic characteristics for the severity of illness and disease outcome than CRP. Zhang et al. [28] have shown that in all patients with COVID-19, SAA levels were increased, and there were statistically significant differences between mild and severe cases.

Our results showed that IL-6 is a helpful predictor of the development of more severe disease wasconsistent with previous studies [19] [22] [29]. Santa Cruz et al. [30] showed that IL-6 correlates well with the development of respiratory failure and survival. In their study, the kinetics of IL-6 levels is fundamental because IL-6 levels could be just temporarily raised. All this could help with early discrimination between survivors and non-survivors and can have therapeutic implications. ROC curve analysis for IL-6 in our study showed good classification performance for severe disease, hospitalization, and survival, outperforming all other APRs investigated in the study. Sabaka et al. [19] had similar results in their study, where IL-6 was the best predictor for oxygen requirement among APRs, with an AUC of 0.911. These findings could be explained by the fact that IL-6 levels rise before other APRs and that secretion of many APRs is IL-6-dependent.

In most of our patients, PCT levels were observed to be below the reference range and also below 0.25 μg/L, which is consistent with previous studies [13] [16] [21]. Based on our and previous studies,significant PCT elevations are rarely seen, so this indirectly can be used as a surrogate marker that empiric antibiotics are not needed in most, if not all, ambulatory care patients with SARS-CoV-2 infection. Even at these low levels, slight variations of PCT concentration were proven useful in predicting severe disease and fatal outcomes in our study. Based on ROC curve analysis, when compared to SAA, PCT had better specificity for predicting death, but the sensitivity was lower than in SAA. The performance of PCT was poorer compared to SAA for predicting disease severity and hospitalization. In the Tong-Minh et al. [31] study, PCT has 95% specificity for hospital admission, which is much higher than in our cohort of patients even when we used a cut-off value of 0.25 as in their study (specificity was 89%, but very poor sensitivity of 6%). Partially, this difference could be explained by a greater number of patients included in the study and a higher number of patients with severe diseases included in the study. It should be remembered that PCT baseline levels are influenced by preexisting chronic diseases, such as chronic kidney disease [32]. Thus, in those patients, during COVID-19, PCT should be interpreted with caution. During COVID-19, in most patients with a non-complicated disease, the PCT value remains within the reference range. Any significant increase in its levels could reflect the development of a severe disease or bacterial superinfection [33].

During COVID-19 infection, ferritin can mediate immune dysregulation via pro-inflammatory effects, contributing to the development of the cytokine storm [34]. Our findings showed that ferritin levels in men and women correlate positively with disease severity and are more elevated in hospitalized patients. This is consistent with more pronounced inflammation in persons with more severe disease. Interestingly, there was no difference between survivors and non-survivors in median ferritin levels. Also, ROC curve analysis showed that AUC for ferritin has no classification performance for death, which is consistent with the findings of Para et al. [15], while in the study of Lino et al. [35], it has fair performance. However, we must remember that both studies included only hospitalized patients. For hospitalization and severe disease, the AUC of ferritin showed fair classification performance. Carubbi et al. [36] found that ferritin levels are not associated with a worse prognosis but correlate with the extensiveness of lung involvement. Compared to SAA, ferritin had lower prognostic utility in our study, which is consistent with previous studies [37] [38]. An interesting study by Abdelhakam et al. [37] showed that a combination of SAA and ferritin improved the performance characteristics of SAA by increasing AUC from 0.928 to 1.000 and sensitivity from 98.5 to 100%.

Fibrinogen levels slightly and slowly rise during the acute-phase reaction. However, even this slight increase was proven in previous studies to correlate with inflammation extensiveness and disease severity in COVID-19 patients [39] [40]. We had a similar observation in our patients, where fibrinogen levels were significantly higher in hospitalized patients and patients with moderate and severe disease. However, interestingly, there was no difference in median values between survivors and non-survivors, which is consistent with the study of Long et al. [40] but not in the study of Siu et al. [39], so the data are conflicting. An intriguing paper by Thachil et al. [41] states that fibrinogen can be protective in COVID-19 infection through inflammatory response regulation independently of the clotting function. Furthermore, the ability of fibrinogen in high levels to saturate Mac-1 receptor for double-strained ribonucleic acid (dsRNA), therefore reducing harmful effects from the virus.

COVID-19-associated coagulopathy is now a well-known entity [42], so the elevation of D-dimervalues during COVID-19 is relatively common. Our study showed that more than half patients had elevated D-dimer levels which is in accordance with the study of Yormaz et al. [10]. Even one-third of patients with mild disease in our cohort have elevated levels of D-dimer, which can be interpreted as low-level activation of the coagulation cascade and asymptomatic coagulopathy. Also, hospitalized patients had higher median values of D-dimer, which is consistent with disease severity in this group of patients. We observed that D-dimer levels were progressively higher with an increase in disease severity, similar to previous studies [11] [12] [29]. Sayit et al. [12] reported that D-dimer is a good marker of pneumonia severity, and their study showed that D-dimer is elevated without obvious thromboembolism. Besides a good correlation with disease severity and hospitalization, based on ROC curve analysis in our study, D-dimer failed to show any valuable addition for assessing COVID-19 patients in ambulatory care settings and, as such, should be used with caution.

Albumin levels were normal in most of our patients. However, even with albumin levels within the reference range, there was a statistically significant difference in median levels between disease severity groups, hospitalized and non-hospitalized patients, and survivors and non-survivors. These findings were consistent with previous studies [17] [43] [44]. In their study, Huang et al. [45] found that low albumin can predict mortality in COVID-19 patients. In the study by Zhang et al. [46], it is found that albumin substitution in patients with more severe diseases may improve survival. When used in an outpatient setting, Turcato et al. [17] found that albumin levels < 35 g/L were independent risk factors for both severe infection and fatal outcome at 30 days, which was almost identical to our findings.

It is shown that transferrin is a good marker for COVID-19 severity [18] [47] [48]. A study by Yadav etal. [47] showed a trend for lower transferrin concentration with increasing disease severity, as shown in our study. However, contrary to our findings, no significant difference existed between survivors and nonsurvivors. This could be explained by the fact that their study included only hospitalized patients, which is supported by similar findings in the study by Shah et al. [48]. Considering poor classification performance for both severe illness and hospitalization and only fair performance for survival based on our analysis, transferrin is not a reliable marker to use in everyday practice for ambulatory care COVID-19 patients assessment compared to other APRs investigated.

## Conclusion

Although our findings should be validated before SAA levels can be used to guide clinical decision-making about the management of COVID-19 patients, our study demonstrated that SAA and otherAPRs could be valuable tools to use in everyday practice. When combined with good history, physical examination, and other diagnostic modalities during the evaluation of SARS-CoV-2-positive patients in an outpatient setting, SAA levels significantly correlate with the severity of COVID-19. As such, SAA levels may serve as an early warning sign of an unfavorable course and more severe disease, and also it could help physicians decide which patients would need more frequent follow-up visits. We found a significant positive correlation between SAA and other parameters for disease severity, hospitalization, and death. Our study found that SAA has a high sensitivity in detecting patients who may have more severe manifestations of SARS-CoV-2 infection, need hospitalization, and may not survive. As a result, SAA levels may help make clinical decisions for improved management of COVID-19 patients.

## Dodatak

### Study limitations

Some associations between SAA and other APR levels and different COVID-19 outcomes might not have achieved significance due to some groups’ low number of patients. Also, in our study, most patients had moderate and severe COVID-19, which is not a realistic representation of the whole population where the mild form of the disease is the most common. It should be kept in mind that we had only 11 patients who did not survive, so analyzing the significance of laboratory parameters in this group and between this and other groups can be imprecise and biased. Also, because the study included only one female patient who did not survive, we could not estimate the significance of APRs for survival in female patients. Another limitation of our study could be the unavailability of data about SARS-CoV-2 variants in patients included because different variants may induce different intensities of immune responses and different APR levels in different patient groups.

### Acknowledgments

This work was supported by the Ministry of Education, Science and Technological Development of the Republic of Serbia based on contracts No.175042, No.175036, and No.451-03-68/2020-14/200161. In addition, we recognize the essential contributions of the clinicians from the Clinic for Infectious and Tropical Diseases »Prof. Dr. Kosta Todorović« on patient recruitment and sample collections: Jelena Simić, Jelena Vlasković, Martina Jug, Ankica Vujović, Ivana Gmizić, Matija Đukić, Vanja Subotić, Uroš Karić, Ivan Rajković, Marko Marković.

### Conflict of interest statement

All the authors declare that they have no conflict of interest in this work.

## References

[1] Zhu N, Zhang D, Wang W, Li X, Yang B, Song J, Zhao X, Huang B, Shi W, Lu R, Niu P, Zhan F, Ma X, Wang D, Xu W, Wu G, Gao G F, Tan W (2020). A Novel Coronavirus from Patients with Pneumonia in China, 2019. N Engl J Med.

[2] 2. World Health Organization (2020). WHO Director-General's opening remarks at the media briefing on COVID-19 - 11 March 2020. https://www.who.int/director-general/speeches/detail/who-director-general-s-opening-remarks-at-themedia-briefing-on-covid-19-11-march-2020.

[3] Zeng H, Ma Y, Zhou Z, Liu W, Huang P, Jiang M, Liu Q, Chen P, Luo H, Chen Y (2021). Spectrum and Clinical Characteristics of Symptomatic and Asymptomatic Coronavirus Disease 2019 (COVID-19) With and Without Pneumonia. Front Med (Lausanne).

[4] García-González P, Tempio F, Fuentes C, Merino C, Vargas L, Simon V, Ramirez-Pereira M, Rojas V, Tobar E, Landskron G, Araya J P, Navarrete M, Bastias C, Tordecilla R (2021). Dysregulated Immune Responses in COVID-19 Patients Correlating With Disease Severity and Invasive Oxygen Requirements. Front Immunol.

[5] Mokhtari T, Hassani F, Ghaffari N, Ebrahimi B, Yarahmadi A, Hassanzadeh G (2020). COVID-19 and multiorgan failure: A narrative review on potential mechanisms. J Mol Histol.

[6] Prabhala S, Sivakoti S, Sahoo B (2021). Utility of acute-phase reactants testing in clinical practice. Indian Journal of Community and Family Medicine.

[7] Letelier P, Encina N, Morales P, Riffo A, Silva H, Riquelme I, Guzmán N (2021). Role of biochemical markers in the monitoring of COVID-19 patients. J Med Biochem.

[8] Khalil R H, Al-Humadi N (2020). Types of acute phase reactants and their importance in vaccination. Biomed Rep.

[9] Sack G H (2018). Serum amyloid A: A review. Mol Med.

[10] Yormaz B, Ergun D, Tulek B, Ergun R, Korez K M, Suerdem M, Kanat F (2020). The evaluation of prognostic value of acute phase reactants in the COVID-19. Bratisl Lek Listy.

[11] Buck M D, Gouwy M, Wang J M, Snick J V, Opdenakker G, Struyf S, Damme J (2016). Structure and Expression of Different Serum Amyloid A (SAA) Variants and their Concentration-Dependent Functions During Host Insults. Curr Med Chem.

[12] Sayit A T, Elmali M, Deveci A, Gedikli O (2021). Relationship between acute phase reactants and prognosis in patients with or without COVID-19 pneumonia. Rev Inst Med Trop Sao Paulo.

[13] Cheng L, Yang J Z, Bai W H, Li Z Y, Sun L F, Yan J J, Zhou C, Tang B (2020). Prognostic value of serum amyloid A in patients with COVID-19. Infection.

[14] Debi H, Itu Z T, Amin M T, Hussain F, Hossain M S (2022). Association of serum C-reactive protein (CRP) and D-dimer concentration on the severity of COVID-19 cases with or without diabetes: A systematic review and meta-analysis. Expert Rev Endocrinol Metab.

[15] Para O, Caruso L, Pestelli G, Tangianu F, Carrara D, Maddaluni L, Tamburello A, Castelnovo L, Fedi G, Guidi S, Pestelli C, Pennella B, Ciarambino T, Nozzoli C (2022). Ferritin as prognostic marker in COVID-19: The FerVid study. Postgrad Med.

[16] Aon M, Alsaeedi A, Alzafiri A, Ibrahim M M, Al-Shammari A, Al-Shammari O, Tawakul M, Taha S, Alherz N, Alshammari J, Albazee E, Alharbi T, Alshammari D, Alenezi Z (2022). The Association between Admission Procalcitonin Level and The Severity of COVID-19 Pneumonia: A Retrospective Cohort Study. Medicina (B Aires).

[17] Turcato G, Zaboli A, Kostić I, Melchioretto B, Ciccariello L, Zaccaria E, Olivato A, Maccagnani A, Pfeifer N, Bonora A (2022). Severity of SARS-CoV-2 infection and albumin levels recorded at the first emergency department evaluation: Amulticentre retrospective observational study. Emerg Med J.

[18] Claise C, Saleh J, Rezek M, Vaulont S, Peyssonnaux C, Edeas M (2022). Low transferrin levels predict heightened inflammation in patients with COVID-19: New insights. Int J Infect Dis.

[19] Sabaka P, Koščálová A, Straka I, Hodosy J, Lipták R, Kmotorková B, Kachlíková M, Kušnírová A (2021). Role of interleukin 6 as a predictive factor for a severe course of Covid-19: Retrospective data analysis of patients from a long-term care facility during Covid-19 outbreak. BMC Infect Dis.

[20] Li H, Xiang X, Ren H, Xu L, Zhao L, Chen X, Long H, Wang Q, Wu Q (2020). Serum Amyloid A is a biomarker of severe Coronavirus Disease and poor prognosis. J Infect.

[21] Chen M, Wu Y, Jia W, Yin M, Hu Z, Wang R, et al (2020). The predictive value of serum amyloid A and C-reactive protein levels for the severity of coronavirus disease 2019. Am J Transl Res.

[22] Liu Q, Dai Y, Feng M, Wang X, Liang W, Yang F (2020). Associations between serum amyloid A, interleukin-6, and COVID-19: A cross-sectional study. J Clin Lab Anal.

[23] 23. World Health Organisation (2021). Clinical management of COVID-19 patients: Living guideline, 23 November 2021. https://apps.who.int/iris/bitstream/handle/10665/349321/WHO-2019-nCoV-clinical-2021.2-eng.pdf.

[24] Demet A F, Karakoyun I, Isbilen B B, Zeytinli A M, Celik E, Dogan K, Duman C (2018). The effects of education and training given to phlebotomists for reducing preanalytical errors. J Med Biochem.

[25] Wang D, Li R, Wang J, Jiang Q, Gao C, Yang J, Ge L, Hu Q (2020). Correlation analysis between disease severity and clinical and biochemical characteristics of 143 cases of COVID-19 in Wuhan, China: A descriptive study. BMC Infect Dis.

[26] Miwata H, Yamada T, Okada M, Kudo T, Kimura H, Morishima T (1993). Serum amyloid A protein in acute viral infections. Arch Dis Child.

[27] Ahnach M, Zbiri S, Nejjari S, Ousti F, Elkettani C (2020). C-reactive protein as an early predictor of COVID-19 severity. J Med Biochem.

[28] Zhang H, Du F, Cao X J, Feng X L, Zhang H, Wu Z X, Wang B, Zhang H, Liu R, Yang J, Ning B, Chen K, Huang Z (2021). Clinical characteristics of coronavirus disease 2019 (COVID-19) in patients out of Wuhan from China: A case control study. BMC Infect Dis.

[29] Velavan T P, Kuk S, Linh L T K, Lamsfus Calle C, Lalremruata A, Pallerla S R, Kreidenweiss A, Held J, Esen M, Gabor J, Neurohr E M, Shamsrizi P, Fathi A, Biecker E (2021). Longitudinal monitoring of laboratory markers characterizes hospitalized and ambulatory COVID-19 patients. Sci Rep.

[30] Santa Cruz A, Mendes-Frias A, Oliveira A I, Dias L, Matos A R, Carvalho A, et al (2021). Interleukin-6 is a biomarker for the development of fatal Severe Acute Respiratory Syndrome Coronavirus 2 pneumonia. Front Immunol.

[31] Tong-Minh K, van der Does Y, Engelen S, de Jong E, Ramakers C, Gommers D, van Gorp E, Endeman H (2022). High procalcitonin levels associated with increased intensive care unit admission and mortality in patients with a COVID-19 infection in the emergency department. BMC Infect Dis.

[32] Level C, Chauveau P, Delmas Y, Lasseur C, Pellé G, Peuchant E, Montaudon D, Combe C (2001). Procalcitonin: A new marker of inflammation in haemodialysis patients?. Nephrol Dial Transplant.

[33] Lippi G, Plebani M (2020). Procalcitonin in patients with severe coronavirus disease 2019 (COVID-19): A meta-analysis. Clin Chim Acta.

[34] Banchini F, Cattaneo G M, Capelli P (2021). Serum ferritin levels in inflammation: A retrospective comparative analysis between COVID-19 and emergency surgical non-COVID-19 patients. World J Emerg Surg.

[35] Lino K, Guimarães G M C, Alves L S, Oliveira A C, Faustino R, Fernandes C S, Tupinambá G, Medeiros T, Silva A A D, Almeida J R (2021). Serum ferritin at admission in hospitalized COVID-19 patients as a predictor of mortality. Braz J Infect Dis.

[36] Carubbi F, Salvati L, Alunno A, Maggi F, Borghi E, Mariani R, Mai F, Paoloni M, Ferri C, Desideri G, Cicogna S, Grassi D (2021). Ferritin is associated with the severity of lung involvement but not with worse prognosis in patients with COVID-19: Data from two Italian COVID-19 units. Sci Rep.

[37] Abdelhakam D A, Badr F M, Abd El Monem Teama M, Bahig Elmihi N, El-Mohamdy M (2022). Serum amyloid A, ferritin and carcinoembryonic antigen as biomarkers of severity in patients with COVID-19. Biomed Rep.

[38] Liu S L, Wang S Y, Sun Y F, Jia Q Y, Yang C L, Cai P J (2020). Expressions of SAA, CRP, and FERR in different severities of COVID-19. Eur Rev Med Pharmacol Sci.

[39] Sui J, Noubouossie D F, Gandotra S, Cao L (2021). Elevated Plasma Fibrinogen Is Associated With Excessive Inflammation and Disease Severity in COVID-19 Patients. Front Cell Infect Microbiol.

[40] Long W, Yang J, Li Z, Li J, Chen S, Chen D, Wang S, Li Q, Hu D, Huang J, Zeng W, Guo L, Wu X (2021). Abnormal Fibrinogen Level as a Prognostic Indicator in Coronavirus Disease Patients: A Retrospective Cohort Study. Front Med (Lausanne).

[41] Thachil J (2020). The protective rather than prothrombotic fibrinogen in COVID-19 and other inflammatory states. J Thromb Haemost.

[42] Conway E M, Mackman N, Warren R Q, Wolberg A S, Mosnier L O, Campbell R A, Gralinski L E, Rondina M T, van de Veerdonk F L, Hoffmeister K M, Griffin J H, Nugent D, Moon K (2022). Understanding COVID-19-associated coagulopathy. Nat Rev Immunol.

[43] Kheir M, Saleem F, Wang C, Mann A, Chua J (2021). Higher albumin levels on admission predict better prognosis in patients with confirmed COVID-19. PLoS One.

[44] Acharya R, Poudel D, Bowers R, Patel A, Schultz E, Bourgeois M, Paswan R, Stockholm S, Batten M, Kafle S, Lonial K, Locklear I (2021). Low Serum Albumin Predicts Severe Outcomes in COVID-19 Infection: A Single-Center Retrospective Case-Control Study. J Clin Med Res.

[45] Huang J, Cheng A, Kumar R, Fang Y, Chen G, Zhu Y, Lin S (2020). Hypoalbuminemia predicts the outcome of COVID-19 independent of age and comorbidity. J Med Virol.

[46] Zhang L, Yu W, Zhao Y, Chen X, Wang P, Fan X, Xu Z (2022). Albumin Infusion May Improve the Prognosis of Critical COVID-19 Patients with Hypoalbuminemia in the Intensive Care Unit: A Retrospective Cohort Study. Infect Drug Resist.

[47] Yadav D, Pvsn K K, Tomo S, Sankanagoudar S, Charan J, Purohit A, Nag V, Bhatia P, Singh K, Dutt N, Garg M K, Sharma P, Misra S, Purohit P (2022). Association of iron-related biomarkers with severity and mortality in COVID-19 patients. J Trace Elem Med Biol.

[48] Shah A, Frost J N, Aaron L, Donovan K, Drakesmith H, Mckechnie S R, Stanworth S J (2020). Systemic hypoferremia and severity of hypoxemic respiratory failure in COVID-19. Crit Care.

